# Lower limbs inter-joint coordination and variability during typical Tai Chi movement in older female adults

**DOI:** 10.3389/fphys.2023.1164923

**Published:** 2023-05-02

**Authors:** Jianmin Zhao, Wei Han, Huiru Tang

**Affiliations:** ^1^ School of Competitive Sport, Shandong Sport University, Jinan, China; ^2^ Digital-Based Performance Training Laboratory, Shandong Sport University, Jinan, China; ^3^ Child Rehabilitation Department, Linyi Maternal and Child Healthcare Hospital, Linyi, China

**Keywords:** older adults, Tai Chi, inter-joint coordination, continuous relative phase, phase plot

## Abstract

**Purpose:** This study aimed to investigate the lower limb inter-joint coordination and variability during Tai Chi movements compared with normal walking in older adults.

**Methods:** A total of 30 female Tai Chi practitioners (70.9 ± 5.2 years) were recruited in this study. Herein, each participant performed three trials of the normal walking and Tai Chi movements. The lower limb kinematics data were collected with Vicon 3D motion capture system. The continuous relative phase (CRP) includes both spatial and temporal information of two adjacent joints, which was calculated to assess the inter-joint coordination of lower limbs. Coordination amplitude and coordination variability were assessed with mean absolute relative phase (MARP) and deviation phase (DP). MANOVOA was used to analyze inter-joint coordination parameters between different movements.

**Results:** The CRP values of hip-knee and knee-ankle segments in the sagittal plane of the Tai Chi movements changed frequently. The MARP values of the hip-knee (*p* < 0.001) and knee-ankle segments (*p* = 0.032) as well as the DP values of the hip-knee segment (*p* < 0.001) were significantly lower in Tai Chi than in normal walking.

**Conclusion:** More consistent and stable inter-joint coordination patterns of Tai Chi movements found in this study may be one of the critical factors that Tai Chi could be a suitable coordinated exercise for older adults.

## 1 Introduction

Falls are a major health problem for older adults worldwide. The incidence and number of falls in elderly women were about twice as high as those in males in the United States (Blazewick et al., 2018). Falls can result in severe injuries, particularly in postmenopausal female adults with a risk of osteoporosis, such as soft tissue injuries, craniocerebral injuries, as well as fractures, and death among older adults (Mateen and Király, 2016). Losing balance is one of the most frequent cause of falls in the elderly (Fabio et al., 2004). Falls prevention requires precise posture control stability, which can be highly related to inter-joints coordination patterns while maintaining body balance (Hsu et al., 2014). However, inappropriate control of the joints of the locomotor system may contribute to body imbalance which may place an individual at risk of falls (Hsu et al., 2016). Therefore, interventions are needed to help the elderly develop strategies for improved inter-joint coordination, particularly female adults.

Thus, Tai Chi is a promising method. As a low-speed, low-impact exercise, Tai Chi is a traditional Chinese exercise that is popular among older adults. In addition, Tai Chi is beneficial for retaining or regaining proper balance and coordination for older people (Kuo et al., 2021). A six-week Tai Chi intervention can improve functional movement coordination, such as placing, turning, and displacing (Wu et al., 2004; Burschka et al., 2014). Given the variable movement velocity between two joints or segments, the kinematic and kinetic analyses of single joints may be insufficient to reveal the coordination characteristics of limbs (Chiu et al., 2010) Inter-joint coordination, which is used to assess the relationship between angular positions and the velocities of two joints, can capture the underlying multiple-joint coordination dynamics in motor tasks to overcome insufficient single-joint coordination (Chiu and Chou, 2012). Therefore, understanding the inter-joint coordination of multiple joints also provides insightful information about postural control and fall prevention in human motion (Mehdizadeh et al., 2015).

Continuous relative phase (CRP) is a commonly used technique to investigate movement coordination. CRP is derived from the angular velocity–angle phase diagram of two joints or segments changing with time, and it is associated with information about afferent joint receptors (Chiu and Chou, 2012). The magnitude and variability of CRP curves are assessed by calculating the mean absolute relative phase (MARP) and deviation phase (DP). In crossing obstacles, Lu et al. observed that during the swing phase, the DP values of the leading limb were smaller than those of the trailing limb; however, the coordination of the leading limb was more stable than that of the trailing limb (Lu et al., 2008). Moreover, limbs with low DPs value show adaptability to environmental changes and have a stable coordination pattern during obstacle crossing of the elderly, concussed, and bilateral osteoarthritis groups (Chiu et al., 2013).

However, the multi-joint coordination of lower limbs is lacking during typical Tai Chi movements, thus more scientific research is needed to understand the basic mechanisms and principles of how Tai Chi works. Therefore, this investigation aimed to describe and quantify the lower limb inter-joint coordination and coordination variability during Tai Chi movements in older adults and explain the beneficial effect of Tai Chi on coordination and proprioception compared with normal walking. For comparison, the normal walking gait is chosen because it is the most common gait pattern in daily activities in elderly populations. In addition, the brush knee and twist step (BKTS) is a typical basic Yang-style Tai Chi movement and continuous forward gait, with gait phases similar to normal walking (Li et al., 2019). It was hypothesized that (1) more tightly coordinated inter-joint coordination dynamics occur during Tai Chi movements than during walking; (2) Tai Chi movements have less variable inter-joint coordination.

## 2 Materials and methods

### 2.1 Participants

A total of 30 healthy female Tai Chi practitioners (age: 70.9 ± 5.2 years, height: 161.4 ± 6.1 cm, body weight: 62.6 ± 8.7 kg, and Tai Chi experience 12.0 ± 5.1 years) were recruited in this study. Inclusion criteria are as follows (Li and Law, 2018): (1) aged 65 years or over; (2) no lower limb injuries; (3) can walk independently and complete the test; and (4) exercise duration of more than 5 years (Song et al., 2017; dan et al., 2018). Meanwhile, exclusion criteria are as follows: inability to follow instructions, heart conditions, joint replacements in the lower extremities, arthritis, visual impairments, vestibular disorders, or any neuromuscular problems preventing the participants from meeting the project requirements. All participants signed an informed consent agreeing to participate. This study has been approved by the Ethics Committee of Shandong Sport University (No. 2017103).

With a sample size of 30, the power of all statistical comparisons in this study was no less than 0.80. It was reported that 15–20 participants were sufficient in estimating the reliability of a quantitative variable (Fleiss, 1986).

### 2.2 Apparatus

**FIGURE 1:**
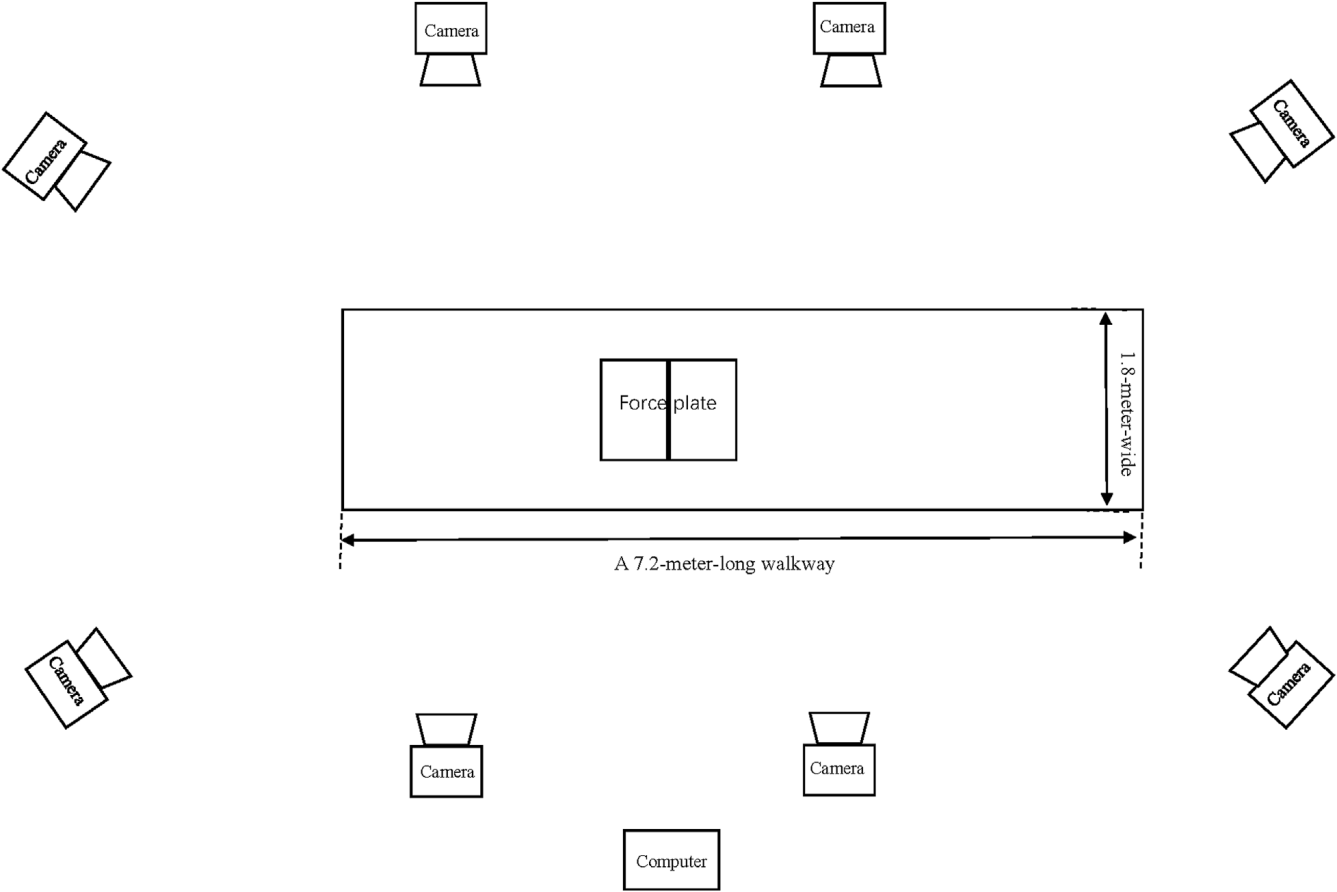
Demonstrations of experimental site.

A walkway was constructed for data collection (Figure 1). Two force plate (Kistler, 9287BA, Winterthur, Switzerland) were embedded into the walkway, and the ground reaction force was collected at 1,000 Hz (Song et al., 2017). The height of the force plate is consistent with the walking path. The kinematics data of lower limbs were collected with an 8-camera Vicon motion capture system (Vicon, Inc., Oxford, UK) at a sampling frequency of 100 Hz (Song et al., 2017). The force and kinematic data collection were synchronized using the Vicon system.

### 2.3 Data collection

The participants’ anthropometric data were collected before the tests. Herein, an experienced research lab technician and participants were blinded to conduct data collection. Then, each participant was asked to wear a tight black uniform and shoes provided by the laboratory. A total of 41 infrared reflective markers with a diameter of 14 mm were attached to the skin or clothes of each participant with double-adhesive tapes. Afterward, before data collection, the participants were instructed to perform 5 min warm-up to familiarize themselves with the experimental set.

**FIGURE 2:**
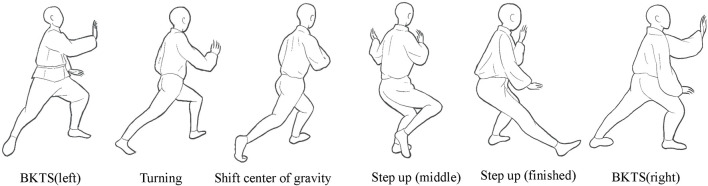
Graph for brush knee and twist step.

The foot movements during the BKTS are described as follows: forward movement, the right foot makes contact with the ground first, the left foot then steps forward, and the right foot leaves the ground at the end (Figure 2). Then, the participants were instructed to perform randomly-assigned tasks independently at a self-selected pace during the experiment. Each subject was asked to practice three times and adjust the starting point of each gait so that the subject’s dominant leg could consistently and naturally contact one force plate at the beginning and another force plate at the end of a complete gait cycle. Once ready, each subject performed three successful trials of each movement (Mao et al., 2006). A successful trial was considered if the subject stepped correctly on all the force plates with a natural and smooth movement, complete gait cycle, and no loss of markers. This was determined by the investigators who observed the subject’s performance. If a trial was not valid, it was discarded in data collection and not used for the subsequent statistical analyses.

### 2.4 Data processing

As determined through a kicking ball test, the right leg was the dominant limb among all participants. In addition, the left and right cycles have quasi-identity during most Tai Chi movements (Mao et al., 2006). Therefore, only the data from the dominant leg was selected for analysis. Besides, given that the kinematic changes of lower extremity joint associated with walking and age-related primarily occur in the sagittal plane (Huang et al., 2020), we only examined the sagittal plane inter-joint coordination between the hip and knee and between the knee and ankle. The gait cycle was defined from the right foot landing on the ground to the same foot landing again for the Tai Chi movement and normal walking. Then, the kinematic parameters of one gait cycle for lower limbs were analyzed, and each gait cycle was normalized to 100 equal time intervals.

#### 2.4.1 Phase plot

The phase portrait for each joint throughout a gait cycle was generated by plotting the normalized angular positions (θ) (*x*-axis) and velocities (ω) (*y*-axis). Angular positions and velocities were normalized using the following equations:

$$\theta_{i}=\frac{2\times \left[\theta_{i}-\min\left(\theta_{i}\right)\right]}{\max\left(\theta_{i}\right)-\min\left(\theta_{i}\right)}-1$$

$$\omega_{i}=\frac{\omega_{i}}{\max\left|\omega_{i}\right|}$$

where θ_i_ and ω_i_ are the angular position and velocity for each data point during a gait cycle, respectively. Such normalization defined the angular positions and velocities between 1 and -1 along both dimensions of the phase plane and minimized individual differences in amplitude and frequency (Tung-Wu et al., 2008). Phase angles were calculated using the following equation at each time point:

$$\varphi =\tan^{-1}\left(\frac{\omega }{\theta }\right)$$

#### 2.4.2 Continuous relative phase (CRP)

The CRP, which identifies the coordination between two adjacent joints, was obtained by subtracting the phase angle of a distal joint from that of a proximal joint (φhip-knee, φknee-ankle) (Lamb and Stöckl, 2014). A positive value indicates that the proximal joint leads to the distal, and *vice versa* (Lamb and Stöckl, 2014). If the Relative Phase Angle (RPA) is close to 2π (0°or ±360°), the two joints are moving in a similar fashion or phase. Meanwhile, the RPA is close to π (180°), the two joints are moving in an opposite fashion or out of phase.

#### 2.4.3 Coordination variability

Mean absolute relative phase (MARP) was calculated by averaging the absolute values of the ensemble curve points for the tasks (Stergiou et al., 2001):

$$MARP=\frac{\sum_{i=1}^{P}\left|\varphi \right|}{P}$$

where P is the number of points in the periods. Functionally, a low MARP value indicates a more in-phase relationship between the action of two joints or segments for a task and a given subject (Ippersiel et al., 2021).

The deviation phase (DP) was calculated by averaging the standard deviations of the ensemble RPA curve points for the tasks (Stergiou et al., 2001; Hein et al., 2012):

$$DP=\frac{\sum_{i=1}^{P}SD}{P}$$

Functionally, a low DP value indicates a stable relationship between the action of two joints or segments for a task and a given subject (Ippersiel et al., 2021).

### 2.5 Statistical analysis

The average values of three successful trials were used for statistical analysis. The multivariate analysis of variance (MANOVA) was used to compare the inter-joint coordination parameters between Tai Chi movements or normal walking. The significance level was set at 0.05. All the statistical analyses were performed with SPSS software 20.0 (IBMS, NY, USA).

## 3 Results

**FIGURE 3:**
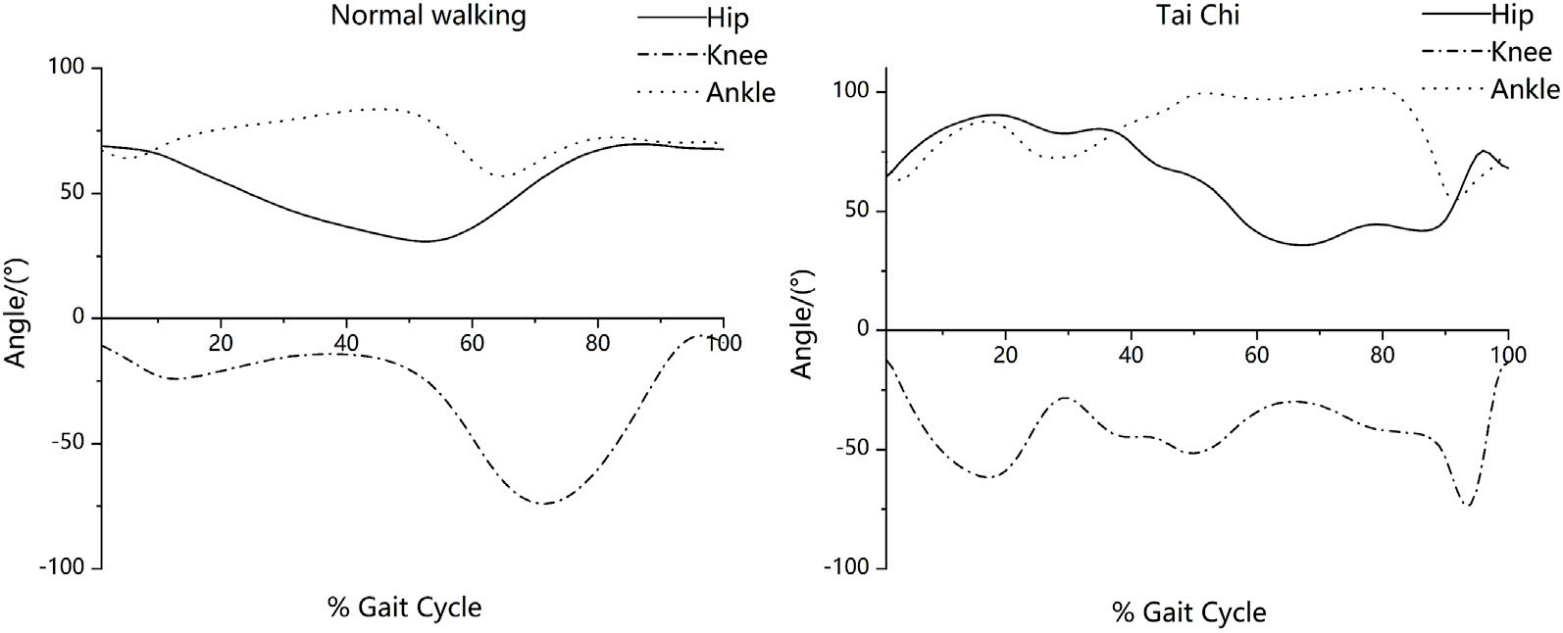
Angles of lower limb joints in the sagittal plane for normal walking and Tai Chi movements.

**FIGURE 4:**
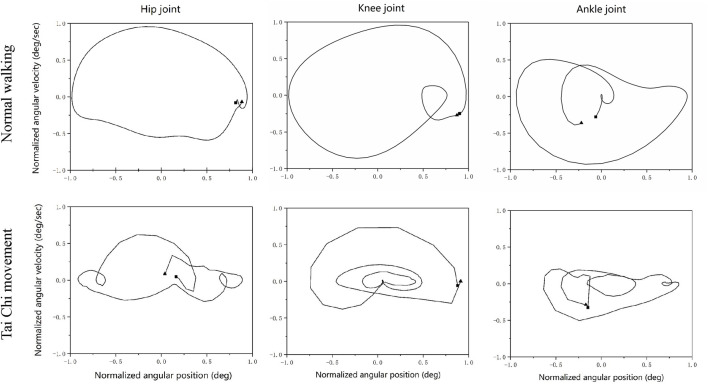
Ensemble-averaged phase plots of the hip, knee, and ankle of the lower limb in the sagittal plane for normal walking and Tai Chi movements. The longitudinal axis is normalized angular velocity and the horizontal axis is a normalized angle.

The flexion and extension angles for the joints changed more frequently in Tai Chi movements as compared with normal walking (Figure 3). As shown in Figure 4, the ensemble-averaged phase plots of the lower limb joints during normal walking and Tai Chi. During normal walking, the hip, knee, and ankle trajectories were all in form of nearly closed and period circles, whereas those for the Tai Chi movements had a more complex form of trajectories with varying amplitudes.

**FIGURE 5:**
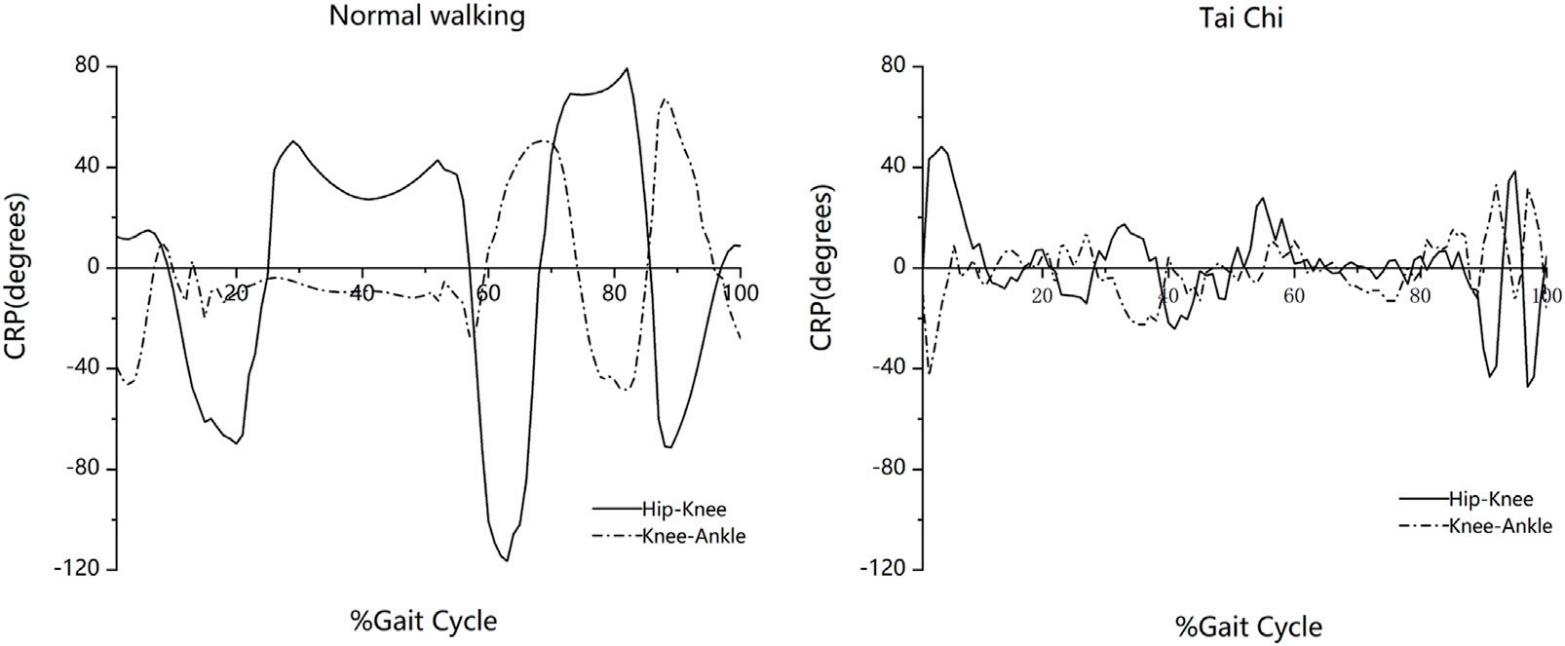
Continuous Relative Phase of the lower limb in the sagittal plane for normal walking and Tai Chi movements.

Figure 5 shows the ensemble-averaged hip-knee (φhip-knee) and knee-ankle (φknee-ankle) CRP of the lower limb joints during normal walking and Tai Chi movements. However, the leading joint between the hip and knee segments and between the knee and ankle segments changed more frequently during Tai Chi as compared with normal walking. During Tai Chi movements, the hip-knee CRP curves showed a W-shape with two negative peaks during the end of gait cycles, whereas the knee-ankle CRP curves showed an M-shape with two positive peaks. Similar shapes were also found for the CRP curves of normal walking, except those of curve changes in Tai Chi movement which lagged behind those of normal walking and the smaller peaks of Tai Chi.

Table 1 provides the MARP and DP values of the CRP curves (hip-knee and knee-ankle) for normal walking and Tai Chi movements. During Tai Chi movements, the MARP and DP values of the hip-knee CRP curves were significantly smaller than those of normal walking (*p* < 0.001). During Tai Chi movement, the MARP values of the knee-ankle CRP were significantly smaller than those of normal walking (*p* = 0.032).

**TABLE 1:** Internal coupling relationship of the sagittal plane of the lower limb (Mean ± SD).

Value (°)	Normal walking	Tai Chi	*p* Value
Hip-Knee MARP	55.44 ± 3.84	36.18 ± 6.11	<0.001
Knee-Ankle MARP	35.54 ± 5.12	32.64 ± 5.09	0.032
Hip-Knee DP	0.64 ± 0.03	0.50 ± 0.06	<0.001
Knee-Ankle DP	0.49 ± 0.06	0.46 ± 0.05	0.069

## 4 Discussion

This study was the first to examine the inter-joint coordination of older adults while performing Tai Chi movements. The present study results exhibited that the inter-joint coordination between the hip and knee and between the knee and ankle moved nearly in phase or synchronized during Tai Chi movement. Furthermore, low variability of hip-knee and knee-ankle coordination was observed during Tai Chi movements. These findings indicate that the Tai Chi movement has better and more stable inter-joint coordination patterns than normal walking. Therefore, the results of this study supported our hypothesis. The result also agrees with previous studies showing that older adults that regularly practice Tai Chi exhibit better postural stability than those who do not (Wu et al., 2004).

In addition, the lower-limb joint-angle changes were more frequent during Tai Chi movement as compared with normal walking. During Tai Chi movement, the present study also found that the phase plot of the three joints of the lower extremities appeared in extra closed circles. Based on previous studies, the phase plots in Figure 4 represent a stable, periodic motion given that velocity and position repeatedly return to a certain value (Lu et al., 2008). Both Tai Chi movement and normal walking are continuous forward movements, but the first one is more complex. During Tai Chi movement, the first step forward, the center of gravity (COP) gradually transitioned to the front limb; then the back limb bent the knee and the front limb extended, the COP shifted to the back limb; and finally turned around, the back limb pushed off, the COP returned to the front limb again. Compared to normal walking, in Tai Chi movements the COP shifted from back to forward by changing the angle of the lower limbs. The motion characteristics may be closely related to the above results.

The frequent changes in joint angle indicate that the elderly need to adjust repeatedly, and accurately control the body’s COP and limb position while performing BKTS. This may help regulate mechanoreceptors’ input-output relationship and induce plasticity changes in the central nervous system (such as the increase of synaptic connection strength) (Ribeiro and Oliveira, 2007). This will also help strengthen signaling pathways and enhance proprioception (Ribeiro and Oliveira, 2007). Proprioception provides feedback on body position and movement that play important role in coordinating movement (Montel, 2019). Moreover, nearly all motile animals rely on proprioceptive feedback to control their bodies (Tuthill and Azim, 2018). The enhancement of proprioceptive conduction function provides more so-called “reflex” pathways for motor output (Tuthill and Azim, 2018), which may allow for more precise movement trajectory and more stable inter-joint coordination patterns of limbs, thereby reducing the risk of falls.

The patterns of the joint phase plot suggest stable inter-segment coordination for each movement. Therefore, the data on inter-joint coordination must be closely investigated for insights into the overall control of the lower extremity during Tai Chi movements. The dominant joint shifts more frequently during Tai Chi movements, thereby indicating changes in the proximal and distal joint-guided motion patterns. At the beginning of the movement cycle, the proximal joint flexes and acts as an active joint to guide motion. Subsequently, hip-knee and knee-ankle coordination gradually changed from out-phase to in-phase to maintain a COP stability. At the end of the movement cycle, the left leg kicks off, and the lower limb flexes to complete an action similar to crossing an obstacle, thereby making joint coordination and posture more difficult to control.

Proximal joints play an important role in postural control, and their dynamic adjustment may increase instability in the body (Rahimzadeh et al., 2020). However, MARP and DP values of hip and knee and knee and ankle CRP curves during the BKTS were lower than normal walking. The smaller the MARP values, the closer the segments are to the in-phase (Ippersiel et al., 2021). Moreover, the smaller the DP values, the lesser the variability, and the more stable the motion (Zhang et al., 2015). This indicates that the motion between the joints of the lower limbs is more synchronized and stable during BKTS, with less independent motion between the joints. This may indicate that older adults adopt more cautious gait strategies when rehearsing knee walking, thereby moving joints more coordinated by frequently adjusting the motion amplitude and speed of adjacent joints. Furthermore, joint coordination plays a crucial role in maintaining gait stability (Ippersiel et al., 2021). This frequent adjustment may be beneficial to optimize the proprioceptive information transmission of the human body and thus promote the formation of adaptive joint coordination patterns between hip-knee and knee-ankle joints. Therefore, BKTS may reflect a coordinated and stable gait control strategy, thereby maintaining posture stability during difficult motor tasks and may help reduce the risk of falls (Mehdizadeh et al., 2015).

However, this study encountered three limitations. First, we disregarded the effects on the trunk and upper limbs. Given that Tai Chi is performed with whole-body segment motions, thus these segments might have been affected. Second, the pattern and variability of inter-joint coordination were only examined in the sagittal plane. Given that the sagittal plane is the primary plane of motion during gait, the inter-joint coordination is expected to be robustly controlled and illustrated. Third, all speeds were self-selected. We intended to examine variations in the inter-joint coordination within a range of self-selected paces and minimize interferences in the participants’ performance.

## 5 Conclusion

Tai Chi movement prompted better adaptability of movement controls and more tightly and stable inter-joint coordination patterns of lower limbs in older adults. This response may reflect a cautious gait pattern, or a functional strategy to help mitigate the risk of falls in older adults. Therefore, Tai Chi exercise may provide a unique way of coordination training, particularly for the coordination between the segments, which may contribute to postural control and thus play a role in preventing falls.

## Data Availability

The raw data supporting the conclusions of this article will be made available by the authors, without undue reservation.
